# Expectant Parents’ Preferences for Teaching by Texting: Development and Usability Study of SmartMom

**DOI:** 10.2196/44661

**Published:** 2023-04-18

**Authors:** Jennifer B Murray, Alexander Sharp, Sarah Munro, Patricia A Janssen

**Affiliations:** 1 School of Population and Public Health Faculty of Medicine University of British Columbia Vancouver, BC Canada; 2 Department of Obstetrics and Gynaecology Faculty of Medicine University of British Columbia Vancouver, BC Canada; 3 Centre for Health Evaluation and Outcome Sciences University of British Columbia Vancouver, BC Canada

**Keywords:** pregnancy, pregnant, prenatal, patient education, text message, SMS text messaging, prenatal education, mHealth, evidence-based health care, mobile app, Canada, mobile health, preference, focus group, information need, user need

## Abstract

**Background:**

Prenatal education encourages healthy behavioral choices and reduces rates of adverse birth outcomes. The use of mobile health (mHealth) technologies during pregnancy is increasing and changing how pregnant people acquire prenatal education. SmartMom is an evidence-based prenatal education SMS text messaging program that overcomes barriers to prenatal class attendance, including rural or remote location, cost, stigma among participants, lack of instructors, and cessation of classes during the COVID-19 pandemic.

**Objective:**

We sought to explore perceived information needs and preferences for the content and structure of prenatal education mHealth programs among persons enrolled in or eligible to enroll in SmartMom.

**Methods:**

This was a qualitative focus group study conducted as part of a development and usability study of the SmartMom program. Participants were older than 19 years of age, Canadian residents, fluent in English, and either currently pregnant or pregnant within the last year. We asked open-ended questions about information-seeking behaviors during pregnancy, the nature of the information that participants were seeking, how they wanted to receive information, and if SmartMom was meeting these needs. Focus groups took place via videoconference technology (Zoom) between August and December 2020. We used reflexive thematic analysis to identify themes that emerged from the data and the constant comparison method to compare initial coding to emerging themes.

**Results:**

We conducted 6 semistructured focus groups with 16 participants. All participants reported living with a partner and owning a cell phone. The majority (n=13, 81%) used at least 1 app for prenatal education. Our analysis revealed that “having reliable information is the most important thing” (theme 1); pregnant people value inclusive, local, and strength-based information (theme 2); and SMS text messages are a simple, easy, and timely modality (“It was nice to have that [information] fed to you”; theme 3). Participants perceived that SmartMom SMS text messages met their needs for prenatal education and were more convenient than using apps. SmartMom’s opt-in supplemental message streams, which allowed users to tailor the program to their needs, were viewed favorably. Participants also identified that prenatal education programs were not meeting the needs of diverse populations, such as Indigenous people and LGBTQIA2S+ (lesbian, gay, bisexual, transgender, queer and/or questioning, intersex, asexual, Two-Spirit plus) communities.

**Conclusions:**

The shift toward digital prenatal education, accelerated by the COVID-19 pandemic, has resulted in a plethora of web- or mobile technology–based programs, but few of these have been evaluated. Participants in our focus groups revealed concerns about the reliability and comprehensiveness of digital resources for prenatal education. The SmartMom SMS text messaging program was viewed as being evidence-based, providing comprehensive content without searching, and permitting tailoring to individual needs through opt-in message streams. Prenatal education must also meet the needs of diverse populations.

## Introduction

Mobile health (mHealth) refers to technologies, including apps and text-based solutions that use mobile devices. The proliferation of mobile devices over the last decade has dramatically changed the way in which pregnant people acquire prenatal education [1,2]. The use of mHealth technology during pregnancy is increasing; international studies have reported the prevalence of app use during pregnancy to range from 50% to 75% [3,4]. Prenatal education has been demonstrated to encourage healthy behavioral choices such as appropriate weight management [5,6], antenatal care attendance [7], smoking cessation [8], decreased alcohol consumption, and increased prevalence of breastfeeding [9]. Moreover, the adoption of these behaviors has been associated with reduced rates of cesarean birth [10,11], preterm birth, and low birth weight [12]. However, as reported in the pan-Canadian Maternity Experiences study, only one-third of Canadian women attends prenatal classes [13]. Barriers to prenatal class attendance faced by Canadian parents include geography, socioeconomic status, age, education, and Indigenous identity [14,15]. Although Indigenous mothers in Canada report widespread use of Google, Facebook, and health-related apps during pregnancy, mothers distrusted the information as it did not align with what they had received from health care providers [16].

SMS text messaging, as a specific mHealth technology, has been shown to change health behaviors [17], including smoking cessation [18,19], weight loss [20], improved physical activity [21], and stress management in nonpregnant populations [22]. A randomized controlled trial (RCT) of the Text4Baby program, developed for low-income communities in the United States, reported higher rates of alcohol reduction in the treatment arm [23]. In South Africa, an RCT showed that SMS text messaging improved attendance for prenatal care, increased rates of vaginal birth after cesarean, and decreased the risk of delivering a low-birth-weight infant [24]. In Zanzibar, a mobile intervention that connected participants to providers decreased perinatal mortality [25]. In Thailand, SMS text messages about uncommon pregnancy symptoms lowered levels of anxiety [26]. A mobile intervention focused on inappropriate weight for gestational age reduced rates of macrosomia in China [27]. A meta-analysis of the effectiveness of mHealth interventions in low- and middle-income countries found that prenatal interventions using SMS text messages were associated with higher rates of breastfeeding at birth [28]. These studies indicate that SMS text messaging programs for pregnant people can improve perinatal health outcomes.

The majority of prenatal mHealth programs for pregnant people have been found to be driven by commercial interests or lay parent groups and lack evidence of effectiveness [29,30]. An overview of 370 apps found through the Google Play Store reported that only 3 were documented as having a scientific board [31].

To our knowledge, there are no SMS text messaging programs that provide pregnant people with comprehensive coverage of prenatal education topics. Unlike apps, texting programs do not require searching through multiple pages or knowledge of what to look for. SMS text messages arrive with content relevant to the stage of pregnancy with no effort on the part of the user.

SmartMom is Canada’s first prenatal education program delivered by SMS text messaging [32]. Participants receive 3 evidence-based texts each week, timed to be salient to their gestational week. Messages contain links to additional web-based sources of information. SmartMom has been endorsed by the Society of Obstetricians and Gynecologists of Canada, and over 13,000 people have enrolled to date. SmartMom messages increase access to those living in rural and remote regions, who might not have easy access to in-person prenatal education. SmartMom is free and accessible to all individuals with access to internet. We experienced an increase in enrollment during the COVID-19 pandemic, which reduced or eliminated access to in-person classes. This study builds on the findings of our usability study that explored women’s preferences for prenatal education [32] and thus informed our decision to design and implement the SmartMom program. In this development and usability study, we sought to extend our examination of the perceived information needs and preferences for the structure of a text-based prenatal education program among those who had been exposed to SmartMom.

## Methods

### The SmartMom Program

The SmartMom Program was initially developed in response to a request from the Northern Health Authority in British Columbia to devise a way to overcome barriers to attendance at prenatal classes, such as remote location, inclement weather and road conditions, low socioeconomic status, a lack of instructors, and stigma perceived by parents who are young or Indigenous. A consortium of University of British Columbia–affiliated academic clinicians and perinatal program leaders from British Columbia health authorities, supported by peer-reviewed funding [33], conducted focus groups with expectant parents in British Columbia to elicit their views on how to proceed in the best possible way [32]. An advisory board was created for SmartMom with representation from all regional health authorities in British Columbia, the BC Ministry of Health, the First Nations Health Authority, Perinatal Services British Columbia, Child Health British Columbia, the BC Ministry of Health, the BC Association of Pregnancy Outreach Programs, CanFASD (Canada Fetal Alcohol Spectrum Disorder Research Network), and KidCare Canada. The development of the program was undertaken with content experts from each of these stakeholder groups with a commitment to iterative user-centered program evaluation and ongoing revision. Persons eligible to enroll in SmartMom are able to read in English, have a healthy singleton pregnancy, and receive maternity care in the province of British Columbia.

### Study Design

We conducted 6 semistructured focus groups with people who were pregnant or recently pregnant to explore their information-seeking behaviors during pregnancy, the nature of the information that they were seeking, how they wanted to receive information, and if SmartMom was meeting or could meet these needs (Multimedia Appendix 1). We selected the focus group methodology to understand shared experiences and perspectives as well as to promote the identification and clarification of views through group discussion [34].

### Setting and Participants

Focus groups were conducted between August and December 2020. Eligible participants were older than 19 years, residents of British Columbia, and able to converse in English. Participants were recruited through (1) unpaid promotion on social media (eg, Twitter and Facebook), (2) brochures at maternity clinics, and (3) snowball sampling by recruited participants. We aimed to achieve diversity in terms of rural or urban residents, parity, and relationship and socioeconomic status.

### Ethics Approval

The University of British Columbia Behavioral Research Ethics Board approved this study (H14-03404). Prior to participation, all participants received a description of the study objectives and procedures and provided written informed consent through a web-based form. Participants were informed that the focus group would be recorded, that they could choose to use their first name or a pseudonym, and whether or not to turn their camera on. All transcripts were deidentified prior to analysis. We provided a CAD $25 (US $18) gift card for participation.

### Study Procedures

Individuals interested in the study contacted our research manager via text messages or email and were provided with details about the study. After an initial discussion, they were sent a link to a digital consent form for review and signature. Participants then completed a demographic questionnaire and reviewed copies of the SmartMom messages. They chose their preferred dates and times for participation, including weekends and evenings. We used videoconference technology (Zoom; Zoom Video Communications, Inc) due to travel restrictions associated with the COVID-19 pandemic.

The focus group discussion was guided by open-ended questions that explored information-seeking behaviors, priority information needs, and current access to information through mobile technology. Each recorded focus group session was transcribed verbatim by a research team member (Emma Martin, BSc) and deidentified prior to analysis. Transcripts were returned to participants for member checking.

### Data Analysis

We undertook reflexive thematic analysis using a constant comparison method [35]. Thematic analysis is an interpretive analysis to identify common themes that emerge based on the phenomenon under investigation and that resonate with the research question [35,36]. The constant comparison method involves reducing the data to codes that describe the data and comparing the initial coding to emerging themes [37]. After an initial read to become familiar with the data, 2 members of the research team (JBM and AS) independently grouped the data based on the similarities and attached a code (open coding). After another reading of the transcripts and a review of the coding, they grouped these codes into categories (axial coding). They then generated themes that summarized these categories (selective coding) [37]. To enhance rigor, both JBM and AS then independently read through the transcripts again to test themes that fit with the coded data. We also tested and refined themes through several team meetings until consensus was achieved. To promote reliability, SM and PAJ reviewed all coding-in-progress and participated in iterative discussions with the analysts to resolve interpretive disagreements, reaching a consensus in both semantics (words used to describe themes) and interpretation (core properties of themes). There were no substantive changes made following member checking. We used qualitative software NVivo (version 12; QSR International) to organize our analysis of the data.

We analyzed demographic data from questionnaires using descriptive statistics. The demographic data supported our understanding of the study population (eg, age and frequency of mobile phone use) and informed our interpretation of the data. We analyzed demographic data using Microsoft Excel (Microsoft Corporation).

### Researcher Reflexivity

Focus group sessions were led by a perinatal epidemiologist (PAJ) with experience moderating focus groups and conducting qualitative research. Research assistants, who were graduate students, Emma Martin, JBM, and an undergraduate AS, cofacilitated the groups. PAJ is the founder and scientific lead of the SmartMom program. PAJ was not involved in the primary analysis of the data but contributed to the review of coding and participated in all iterative discussions of the analysis.

The aim of reflexive thematic analysis is to be recursive, where the analysis goes back and forth between phases of coding and reviewing themes. To achieve this, all authors engaged in reflexivity throughout the analysis and writing of the results [38]. Throughout the analysis, JBM and AS discussed the influence of their own knowledge of pregnancy experiences on their generation of codes and themes. All study team members considered the influence of age, gender identities, and racial or ethnic backgrounds on our interpretation of data. We discussed the influence of these social locations on all stages of the analytic process in our team meetings.

## Results

### Participants

The 16 participants resided in British Columbia, except for 1 participant who resided in eastern Canada (Table 1). Half (n=7, 54%) were aged 30-39 years old. All were born in Canada and reported living with a partner. Of this, 77% (n=10) reported household annual revenue of CAD $75,000 (US $54,879) or more and having a university degree (n=11, 85%). All reported owning a cell phone, using SMS text messaging, using apps, and browsing the internet using their mobile device (Table 2). Of those who discussed phone use (n=10), 80% (n=8) used their phone for 5-6 hours a day or more, while 20% (n=2) used it for 2-4 hours a day. The participants used at least 1 app for prenatal education in 81% (n=13) of the cases. Prior to the focus groups, all participants had an opportunity to review the SmartMom SMS text messaging, and 44% of participants had enrolled in SmartMom during pregnancy. All participants were pregnant or recently pregnant during the COVID-19 pandemic. Themes generated through analysis are presented in Textbox 1 and summarized with representative quotes below.

**Table 1:** Characteristics of study participants (N=13)^a^.

Characteristics			Participants, n (%)
**Age (years)**			
	20-29	5 (38)	
	30-39	7 (54)	
	>40	1 (8)	
**Best description of living area**			
	No answer	1 (8)	
	Rural area	5 (38)	
	Suburban area	2 (15)	
	Urban area	5 (38)	
**Number of children**			
	0	2 (15)	
	1	7 (54)	
	2	4 (31)	
**Household annual income**			
	<CAD $35,000 (US $25,610)	2 (15)	
	CAD $35,000-CAD $75,000 (US $25,610-US $54,879)	1 (8)	
	>CAD $75,000 (US $54,879)	10 (77)	
**Education**			
	High school	1 (8)	
	College or technical trade	1 (8)	
	University degree	11 (85)	
**Maternal status**			
	Adoptive parent	1 (8)	
	Biological parent	11 (85)	
	Currently pregnant	1 (8)	
**Occupation**			
	Business	1 (8)	
	Childcare or teaching	2 (15)	
	Health care	3 (23)	
	Services or recreation	5 (38)	
	Stay at home parent	1 (8)	
	Student	1 (8)	
**BC^b^ health authority**			
	Fraser health	2 (15)	
	Northern health	6 (46)	
	Vancouver coastal health	2 (15)	
	Vancouver island health	2 (15)	
	Other province	1 (8)	

^a^Among 16 participants, 13 completed a demographic questionnaire.

^b^BC: British Columbia.

**Table 2:** Mobile phone usage (N=16).

		Participants, n (%)
**Hours per day**		
	2-4	2 (13)
	5-6	8 (50)
	Did not discuss	6 (38)
**Number of apps used during pregnancy**		
	None	1 (6)
	1	4 (25)
	2	5 (31)
	3+	4 (25)
	No answer	2 (13)
**Data plan**		
	Limited	3 (19)
	Unlimited	9 (56)
	No answer	4 (25)

**Themes generated from thematic analysis.**

1. Having reliable information is the most important thing

- Seeking reliable information
- Constant Googling
- Seeking social interactions
- Connecting with information through social media
- Consequences of the COVID-19 pandemic

2. Valuing inclusive, local, and strength-based information

- Valuing inclusive content
- Importance of local context
- Using social connections to “keep positive”
- Strength-based content

3. It was nice to have that [information] fed to you

### Theme 1: “Having Reliable Information is the Most Important Thing”

Participants engaged in a process of seeking and assessing reliable information through Google and social media platforms to address specific pregnancy-related questions, signs, and symptoms. This behavior was amplified as a result of COVID-19 pandemic restrictions on antenatal visits and in-person classes.

#### Seeking Reliable Information

All participants spoke about finding resources that were trustworthy: “Having reliable information is the most important thing [for prenatal education information]” (Participant 5). It was difficult to wade through the large volume of prenatal education available digitally and figure out “where the information is coming from” to determine if it was trustworthy (Participant 8). One participant expressed how they could “trust” SmartMom because “it linked back to [university] or health system evidence and it was clear it was not just a Google website” (Participant 5). Many participants followed doctors on social media or consulted their maternity provider to learn how to access reliable information on web-based platforms. The author of such web-based information was a key indicator of trustworthiness.

#### Constant Googling

Most participants were “constantly looking up stuff” (Participant 4) on their mobile devices during pregnancy and in the postpartum period. Looking up signs and symptoms helped provide assurance that their experiences were “normal.” They described “just googling random stuff” in the evening, “googling nonstop” about their symptoms, or being on their phone “a shocking amount.”

#### Seeking Social Interactions

Participants, especially those pregnant for the first time, reported seeking social connections during pregnancy. Nearly all joined a web-based social community (eg, Reddit, Facebook, and blogs) to connect with and learn from other people who were pregnant. While not all participants actively interacted in web-based communities, most benefited from reading about other pregnancy experiences, as one participant described:

It’s mostly about sharing experiences. And a lot of it is lighthearted. But then a lot of it is also, you know, there’s a lot of camaraderie. You have first time moms who have no idea what’s coming next and parents who have more children. They’re comparing pregnancies and sharing what helped them before

Participant 13

Another benefit of social connections was to confirm that pregnancy experiences, such as symptoms, were “really normal” (Participant 13) or not “super weird” (Participant 16).

#### Connecting With Information Through Social Media

Participants told us that social media was an important way for parents to find evidence-based prenatal educational information. “Right now, most people of childbearing years are on apps like TikTok or Facebook or Instagram” (Participant 10). Social media platforms informed participants about how to access prenatal educational information (eg, community pregnancy outreach Facebook pages) in addition to providing specific information. One participant commented on how social media content linked to a trusted source is especially impactful:

There are a lot of dietitians for postnatal that are now doing takeovers [a partnership to post content from someone else’s account] of my doctor’s accounts, their followers get the [dietician’s] information as well

Participant 15

#### Consequences of the COVID-19 Pandemic

The COVID-19 pandemic led to a reduction in in-person prenatal care. This, along with increased reliance on digital prenatal education, amplified stress. “It feels very rushed. I just question if everything’s being checked off” (Participant 11). One participant described how she felt “disconnected” from prenatal providers, “like she had to search out information on [her] own,” and when she had a chance to have an in-person visit, she had to “really remember [her] questions” (Participant 8).

### Theme 2: Valuing Inclusive, Local, and Strength-Based Information

Participants were asked about how mobile apps could be tailored to meet their needs.

#### Valuing Inclusive Content

Participants desired information about a broad diversity of pregnancy experiences:

In my networking group with mamas the whole piece about transgender/transgendered moms and pregnancies, that’s a really big deal, as it should be, because it’s just something that isn’t really talked about that often

Participant 11

Participants also reflected on the lack of resources to support expectant parents who are not themselves the birther: “I haven’t come across anything that I would recommend to a fellow adoptee or a parent trying to adopt or go through surrogacy” (Participant 10). Participants also wanted resources that were appropriate for parents with lower education or literacy, for younger parents, as well as resources with a culturally safe lens for Indigenous parents. Participants also requested content for pregnancy loss; surrogacy; lesbian, gay, bisexual, transgender, queer and/or questioning, intersex, asexual, Two-Spirit plus (LGBTQIA2S+) pregnancy; and fathers.

#### Importance of Local Context

Participants expressed frustration that the most-accessed mobile apps were from the United States and other countries and often provided irrelevant information. “I didn’t like having to filter through and figure out what is relevant. What is Canada and the Canadian reality?” (Participant 15). Participants strongly desired having local information (eg, a list of local resources) available through their mobile app. Participants often found web-based information that was topically appropriate but lacked local relevance, as 1 participant reflected:

I like traditional prenatal classes and especially during COVID, because you couldn’t do it in person. I was looking at videos from the States about what to expect during childbirth. That was information that I would have appreciated from a more local standpoint

Participant 6

#### Using Social Connections to “Keep Positive”

Many found it useful when mobile apps offered a way to connect with other pregnant people and new parents.

I really find it helpful talking to other parents and other moms and hearing really good experiences, too. It kind of helps you keep more positive and not scared

Participant 7

Hearing personal stories made information more relevant and relatable and facilitated comparing pregnancy experiences. Reading about positive pregnancy experiences reassured participants that their own health concerns were common.

#### Strength-Based Content

While participants wanted to be aware of risks in pregnancy, they disliked “fear-based” information (Participant 11). Many sources of web-based prenatal education information evoked anxiety. One participant advised other moms: “Don’t Google, ever” (Participant 14). Another participant reflected: “I had the SmartMom texts all the way through my pregnancy and I really liked that. It helped me feel more comfortable and know what was normal and what was good” (Participant 11). Not having an easy way to contact health professionals to ask questions further exacerbated the anxiety arising from web-based sources of information. “I think it’s really important that information not disempower people” (Participant 4).

### Theme 3: It Was Nice to Have That Information Fed to You

Most participants liked the simplicity and ease of receiving SMS text messages in the SmartMom program. In comparison, participants felt mobile apps and websites did not offer the same functionality or tailoring. They noted that having the SMS text messages delivered to them reassured them that they were not missing important information:

I was always worried that I would miss something that I was supposed to be worried about. So, it was nice to have that, that it’s fed it to you. I thought they were pretty comprehensive.

They also appreciated the opportunity to have optional message streams that were relevant to their pregnancy:

I really liked the ability to opt in and have it tailored. Like, are you over 35? And then having that stream was something that is nice because then it was some assurance that what you’re going to receive is things that you should be worrying about

Participant 15

Most participants agreed that additional prenatal content delivered via an app would be beneficial. Participants told us they would like to have push notifications, pregnancy tracking, downloadable content, and a search function in an app. One participant explained how such functions would support their information needs:

It’s nice if you can have a platform where you can go back and say you got a text like a few weeks ago and you really liked the article and you wanted to go back to it. I don’t know if it would be as easy to go through your messages versus if it’s on an app platform, you can easily find exactly what you’re looking for

Participant 14

Participants also wanted to have the option of connecting to a qualified health care professional through their mobile app.

## Discussion

### Principal Results

Our findings suggest that expectant parents are looking for trustworthy, evidence-based information that is locally relevant, delivered with tailored content, and sent to them automatically rather than requiring them to seek information out. The SmartMom prenatal education program was highly valued, as texting made comprehensive information easy to access in addition to being reliable, relevant, timely, and tailored. This finding was similar to the results of our usability study [32]. Participants spoke about how the opportunity to digitally connect with other pregnant people was a benefit of accessing other people’s stories on web-based platforms. These connections and stories offered reassurance and normalized prenatal experiences [39]. A strength-based approach to messaging was also identified as a potential improvement in other prenatal education messages.

This development and usability study of the SmartMom program builds on the findings of our initial study undertaken to inform program development. Different from Munro et al [32], participants in this study were pregnant or recently pregnant during the COVID-19 pandemic, which led to a dramatic shift away from in-person prenatal appointments. Participants spoke about the necessity of having reliable information about pregnancy given the abundance of information available on web-based platforms. They also spoke about the value of social media in pregnancy, which may reflect changes in the use of social media since 2017. To address preferences for social connections in pregnancy, we are conducting a nurse-moderated forum where SmartMom users will be able to converse and exchange information with each other in real time in a digital environment. To understand the association of SmartMom with birth outcomes, we are conducting a prospective observational study. We recently received funding to conduct a cross-Canada RCT of SmartMom to evaluate its impact on maternal, fetal, and newborn outcomes.

There has been a major shift from in-person to digital prenatal education in the last decade [2]. Public health restrictions due to the COVID-19 pandemic have led to an unprecedented acceleration of this shift [40], and reliance on mobile technology for prenatal education will likely persist. Equitable access to web-based education is thus increasingly important. Similar to our findings, a 2018 study in Canada found substantial gaps in both resources and representation in prenatal resources for members of LGBTQIA2S+ communities, teenagers, and individuals with low literacy, disabilities, low socioeconomic status, and immigrants [41]. In Canada, Indigenous communities and remote communities are especially vulnerable to receiving inadequate prenatal education, which is particularly concerning in view of reports of reduced access to aspects of routine screening, such as ultrasonography [42]. Our work has shown to date that a lack of reliable internet or cellular access disproportionally affects Indigenous people, who often live in the most remote locations [43]. To address this, we are developing an offline app that will store SmartMom messaging on a phone. We are exploring how to implement specific functionality preferences identified in this development and usability study in the offline app, such as the desire to pair a texting program with an app and a search function to quickly find information.

### Strengths and Limitations

This study had several strengths. Our focus group design allowed for a rich discussion of experiences and perceptions as well as the opportunity for clarification and expansion of ideas. Remote participation permitted participation from a cross section of rural, suburban, and urban locations.

There were some limitations. We did not collect data on gender, race, or ethnicity. The majority of participants in this study were educated and economically advantaged, and all had a partner. While our sample lacked a broad diversity of experiences, themes identified in this analysis aligned with answers to open-ended questions by SmartMom participants between 2017 and 2020 as part of the ongoing evaluation of the program [44]. That evaluation (n=970) was undertaken in a diverse group with age, education, and ethnicity, including Indigenous identity, representative of the BC population [44], suggesting that our focus group findings may be generalizable to larger populations.

### Conclusions

While pregnant people routinely use apps for prenatal education, they have concerns about the reliability and comprehensiveness of the content. The SmartMom prenatal education program delivered via text was viewed as an improvement in that it was evidence-based, provided comprehensive content without searching, and allowed for tailoring to individual needs through opt-in message streams.

### Future Directions

This study offers new patient-oriented parameters to evaluate mHealth education programs.

Current offerings of mHealth prenatal education programs do not address the diversity of pregnant populations today, including Indigenous or LGBTQIA2S+ communities. The shift away from in-person prenatal education to digital mobile technology—accelerated by the COVID-19 pandemic—means that investing in mHealth programs that meet the needs of pregnant people is essential to providing effective prenatal education. Equitable access to mHealth education is also imperative to ensure that all pregnant people may benefit from prenatal education programs.

## Appendix

Multimedia Appendix 1 SmartMom focus group interview guide.

## Abbreviations

CanFASD: Canada Fetal Alcohol Spectrum Disorder Research Network
LGBTQIA2S+: lesbian, gay, bisexual, transgender, queer and/or questioning, intersex, asexual, Two-Spirit plus
mHealth: mobile health
RCT: randomized controlled trial

## Data Availability

We have shared our questionnaire, which is cited in the Methods section (Multimedia Appendix 1). The data sets generated and analyzed during this study are not publicly available due to the individual privacy rights of our participants and as outlined to them during the consenting process.
