# Supplementary material for: Expectant Parents’ Preferences for Teaching by Texting: Development and Usability Study of SmartMom
Source: JMIR Form Res. 2023 Apr 18;7:e44661. doi: 10.2196/44661 (PMC10155084; doi:10.2196/44661)
Supplement: Multimedia Appendix 1 [file formative_v7i1e44661_app1.pdf]

Appendix 1

SmartMom Focus Group Interview Guide

Step 1: Introduction (10 minutes)

Check if all have filled out the Consent and Demographic forms
Reminder of options to maintain privacy and confidentiality – Zoom video and nickname is optional

Step 2: Part 1 of focus group interview questions (20 minutes)

RELEVANT INFORMATION & BEHAVIOUR

- In order to best care for you and your baby, what information about pregnancy do you find the most important or relevant?

- Are there particular topics or questions you have about pregnancy that you struggle to find information about?

- How do you gain advice and information about your pregnancy? (Probes: family, Internet, doctor?)
    - If you have not been pregnant before, where would you go to gain this information?
    - How did you get the information? (Probes: Was it handed to you, emailed, did you seek it out yourself?)

Step 3: Part 2 of focus group interview questions (30 minutes)

SMARTMOM & APP FEASIBILITY

Note: SmartMom messages were distributed to study participants.

Brief description of SmartMom to individuals who are not/have not been SmartMom participants:

SmartMom is a free text-messaging service that provides pregnant women with evidence-based information about healthy pregnancy and birth. This information is timed to their stage of pregnancy and also can help direct expectant mothers to local, regional and provincial services and resources.

Text messages are delivered to women free of charge and include information about options, links to recommended resources specific to their community, and encourage women to discuss important issues in pregnancy, childbirth and newborn care with their healthcare provider.

- Have you heard of Smartmom before?
    - How did you learn about SmartMom?

- What would be the best way to let women know about SmartMom? (Probes: Physicians,

friends, social medias?)

- How long do you spend on your phone each day?

- Do you currently use pregnancy apps on your phone?
  - If so, which apps?
  - If you are not pregnant, would you be interested in using pregnancy apps?

- What is good about these apps? Bad?

- Would you sign up for a SmartMom app or recommend it to others? Why/why not?

- If you could change anything about SmartMom or add something to it, what would it be?

- If you were asked, would you be willing to provide your personal health number to health authorities, knowing that it would not be kept in a research database? It would be used to link to your pregnancy outcome data (rates of preterm birth, inadequate fetal growth, inappropriate maternal weight gain) by your Health Authority but returned to researchers without identifiers.
